# Biological properties of bone marrow plasma cells influence their recovery in aspirate specimens: impact on classification of plasma cell disorders and potential bias to evaluation of treatment response

**DOI:** 10.1007/s00277-020-04249-2

**Published:** 2020-09-15

**Authors:** Svitlana Demyanets, Alexandra Kaider, Ingrid Simonitsch-Klupp, Günther Bayer, Almira Subasic, Renate Thalhammer, Harald Esterbauer, Maria T. Krauth, Hermine Agis, Thomas Reiter, Ilse Schwarzinger

**Affiliations:** 1 Department of Laboratory Medicine, Medical University of Vienna, Vienna, Austria; 2 Center for Medical Statistics, Informatics, and Intelligent Systems Section for Clinical Biometrics, Medical University of Vienna, Vienna, Austria; 3 Department of Pathology, Medical University of Vienna, Vienna, Austria; 4 Department of Internal Medicine I, Division of Hematology and Hemostaseology, Medical University of Vienna, Vienna, Austria; 5 Department of Internal Medicine I, Division of Oncology, Medical University of Vienna, Vienna, Austria; 6 Department of Internal Medicine III, Division of Nephrology and Dialysis, Medical University of Vienna, Vienna, Austria

**Keywords:** Plasma cell recovery, Bone marrow aspirate, Bone marrow biopsy, CD56, Cytogenetic aberrations

## Abstract

Methods to estimate bone marrow plasma cells (BMPC) basically include histopathology, cytomorphology, and flow cytometry. The present study compares the outcomes of these methods with special focus on the impact of BMPC-specific characteristics on their recovery by either method. Laboratory reports of diagnostic samples from 238 consecutive patients with suspected or known plasma cell disease were retrospectively analyzed. The median (IQR) proportion of BMPC was 30.0% (15.0–70.0%) by histological review (hBMPC), 7.0% (2.0–16.0%) by smear review (sBMPC), and 3.0% (0.8–10.0%) by flow cytometry (fBMPC). The disparity of results between core biopsy and aspirate smear was enhanced in case of poor quality of the smear, increased BM fiber content, higher grade cell atypia, expression of CD56 (all *P* < 0.0001), the number of cytogenetic aberrations (*P* = 0.0002), and abnormalities of the *MYC* gene (*P* = 0.0002). Conversely, expression of CD19 and a non-clonal plasma cell phenotype were associated with a lower difference between hBMPC and sBMPC (both *P* < 0.0001). The disparity between the percentages of sBMPC and fBMPC was associated with the quality of the smear (*P* = 0.0007) and expression of CD56 (*P* < 0.0001). Our results suggest that the recovery of BMPC in aspirate specimens not only is a matter of sampling quality but also depends on biological cell properties. Aspiration failure due to malignant type features of BMPC may lead to misclassification of plasma cell disorders and represent a bias for the detection of minimal residual disease after therapy.

## Introduction

According to the recently revised criteria for the classification of plasma cell dyscrasias, the percentage of clonal bone marrow plasma cells (BMPC) is a decision-making parameter. First, the presence of at least 10% clonal BMPC is mandatory for separation of plasma cell myeloma from monoclonal gammopathy of undetermined significance (MGUS), and second, the presence of ≥ 60% clonal BMPC has been established as a disease-defining biomarker for symptomatic plasma cell myeloma [1, 2]. Available methods to estimate BMPC basically include histopathology, cytomorphology, and flow cytometry. Histopathologic review of a core biopsy specimen is the most realistic approach to assess the actual in situ status within the BM. Accordingly, architectural anomalies like bone marrow fibrosis or patchy disease can only be detected by histopathology [3]. Plasma cells in core biopsy specimens are usually identified by combining a conventional hematoxylin/eosin stain with specific immunohistochemical stains. BM biopsy specimens have to get decalcified before further manipulation, and therefore, the entire investigation process takes about 3–4 days. By contrast, cytomorphologic review of the aspirate smear is a straightforward procedure that may be performed immediately after BM withdrawal. Plasma cells can easily be identified with panoptic stains, and details on cell morphology are best assessed by cytomorphology [3]. On the other hand, smear preparations do not reflect the entire in situ status, because BM cells are cut loose from the architectural context and may be diluted with concomitantly aspirated peripheral blood. Flow cytometric detection of BMPC is based on their reactivity with plasma cell-specific monoclonal antibody combinations [4]. Abnormal plasma cells are characterized by aberrant surface antigen expression, and clonality is assessed by intracytoplasmic light chain expression. Flow cytometry requires higher aspirate volumes than cytomorphology, which means extended pull out times with higher risk of peripheral blood contamination. Thus, the morphological methods have been shown to be superior in terms of plasma cell recovery and are, therefore, recommended for estimation of diagnostic threshold levels [2, 4–6]. Flow cytometry is essential to characterize aberrant antigen expression at diagnosis and to quantify residual aberrant cells after treatment [4, 6, 7]. In the present study, we compared the percentages of BMPC obtained in parallel by all three methods. Discrepancies of results were evaluated with respect to their particular impact on diagnostic and therapeutic decisions and associated with cell-specific characteristics. Additionally, we investigated whether the method of sample preparation influences the flow cytometric yield of BMPC.

## Methods

### Study design

Cases that had been investigated for estimation and characterization of BMPC were retrospectively identified from the databases of the Department of Pathology, Medical University of Vienna (MUV), and the Department of Laboratory Medicine, MUV. The hematology laboratories of both institutions practice a diagnostic partnership and also provide each other with electronic access to either test results. Between January 2016 and May 2018, bone marrow specimens from 238 patients with suspected or known plasma cell dyscrasias had been investigated by histo- and cytomorphologic reviews as well as flow cytometry. Histological examinations from core biopsy specimens had been performed at the Department of Pathology. Cytomorphologic reviews from aspirate smears as well as flow cytometric and cytogenetic analyses had been performed at the Department of Laboratory Medicine. Referring institutions had included three clinical divisions of the Vienna General Hospital/MUV (Division of Hematology and Hemostaseology, Division of Oncology, both Department of Internal Medicine I, and Division of Nephrology and Dialysis, Department of Internal Medicine III) and various extern hospitals in the Vienna area. Details on the total number of BMPC and their morphologic, immunophenotypic, and cytogenetic features as well as information on the quality of investigated specimens were extracted from the respective laboratory records.

Prompted by the results of this initial study, a further prospective analysis of 40 bone marrow specimens was initiated to evaluate the influence of sample preparation procedures on flow cytometric results.

### Core biopsy

Bone marrow biopsies had been routinely processed after fixation in Schaffer’s solution (formalin-ethanol) and decalcified with EDTA. Two-micrometer sections had been cut from each specimen and stained with H&E, Giemsa, PAS, Gomorri’s reticulin and Napthol-AS-D chloroactetat esterase. For amyloid assessment, a Congo-red stain had been applied using 5-μm thick sections cut from each block and analyzed under polarized light.

Immunohistology had been performed with an automated BOND-III immunohistology stainer using antibodies against CD20 (DAKO, Cat.-Nr. M0755), CD38 (NovoCastra (Leica Biosystems), Cat.-Nr. NCL-L-CD38-290), CD117 (DAKO, Cat.-Nr. A4502), CD56 (NovoCastra (Leica Biosystems), Cat.-Nr. NCL-L-CD56-504), Cyclin-D1 (Neomarkers, Cat.-Nr. RM-9104-S), kappa (DAKO, Cat.-Nr. A0191), and lambda (DAKO, Cat.-Nr. A0193). Kappa and lambda-light-chain expression had been evaluated using a double-staining procedure. The plasma cell content had been estimated as the percentage of the whole cellularity using the CD38 immunohistological stain. Only CD38+ cells with clear-cut plasma-cell morphology had been taken into account for the estimation of the plasma cell population (i.e., scattered lymphocytes with usually weaker CD38-expression than the plasma cells were excluded).

On the basis of the prerequisites outlined by the WHO for the classification of myeloid neoplasms, the quality of biopsies had been graded very good (proper fixation, adequate length, no crush artifacts and/or fragmentation, right angle from the cortical bone with several intratrabecular areas), good (any criteria inadequate), moderate (2 parameters inadequate), and poor (> 2 parameters inadequate) [1].

### Aspirate smear

BM aspirate smears had been prepared from first pull samples. Slides had been stained by a modified Wright technique and the proportion of BMPC had been quantified by counting 500 nucleated cells. In case of significant dilution with peripheral blood (< 15% erythroid and myeloid precursor cells), smears had been classified as not adequate.

### Flow cytometry

#### Materials

Flow cytometric analyses were performed from second pull aspiration samples of heparin-anticoagulated BM. Mononuclear cells (MNC) were isolated using Ficoll (Ficoll-Paque PLUS, GE Healthcare, Uppsala, Sweden) density gradient centrifugation according to the manufacturer’s instructions (Ficoll method). Total white blood cells (WBC) were recovered by the red blood cell (RBC) lysis method. Briefly, BM specimens were incubated for 10 min at room temperature in a tenfold excess of ammonium chlorid solution (IOTest 3; Immunotech SAS), centrifuged for 5 min at 500*g*, and washed twice with phosphate buffered saline (PBS).

In the retrospective cohort, flow cytometric analyses had been performed from MNC preparations. In the prospective cohort, aspirate materials were split and one aliquot was subjected to Ficoll density gradient centrifugation, whereas the other aliquot was processed by the RBC lysis method.

#### FACS analysis

A total of 6 × 10^5^ cells were incubated for 30 min at room temperature with fluorochrome-conjugated antigen-specific monoclonal antibodies according to the supplier’s recommendations. For staining of intracytoplasmatic antigens, cells were permeabilized and fixed with the IntraPrep permeabilization reagents (Beckman Coulter, Miami, FL, USA). The antibody panel consisted of clones CD45-V500-C clone 2D1 (Becton, Dickinson, BD, Cat.-Nr. 655873), CD138-PC5 clone B-A38 (Beckman Coulter, Cat.-Nr. A54191), CD38-PE clone HB-7 (BD, Cat.-Nr. 345806), CD19-APC clone SJ25C1 (BD, Cat.-Nr. 345791), CD56-FITC clone NCAM16.2 (BD, Cat.-Nr. 345811), CD117-PE-Cy7 clone 104D2 (BD, Cat.-Nr. 339217), and CD20-V450 clone L27 (BD, Cat.-Nr. 655872) for cell surface staining and kappa-FITC clone TB28-2 (BD, Cat.-Nr. 644059) and lambda-PE clone 1-155-2 (BD, Cat.-Nr. 642925) for intracytoplasmatic stains. After a final washing step, cells were resuspended in PBS and analyzed on a three-laser FACS Canto II flow cytometer equipped with FACS Diva^TM^ software (Becton Dickinson, San Jose, CA, USA). Between 20,000 and 500,000 events were acquired to recover a significant plasma cell population of at least 100 cells. Plasma cells were identified using a combination of CD138, CD38, and CD45 together with light scatter characteristics and finally quantified by gating on CD138 positive cells with strong expression of CD38 [4]. Results were expressed as proportion of CD138/CD38++ cells among total MNC (Ficoll method) or total WBC (RBC lysis method).The expression profile of CD19, CD56, CD117, and CD20 was tracked on the CD138/CD38++ population. Antigen expression was scored positive for statistical analysis if the respective antigen had been detectable in > 80% of the BMPC population.

### Fluorescence in situ hybridization

CD138+ cells had been isolated from 185 bone marrow samples by MACS separation (Miltenyi Biotec GmbH, Bergisch Gladbach, Germany) according to the manufacturer’s instructions. In the remaining 53 cases, either no material had been sent for cytogenetic workup or the number of sBMPC had been below 2%, which is the laboratory cutoff for the isolation of CD138+ cells.

Analyses had been performed using MetaSystems DNA fluorescence in situ hybridization (FISH) probes (MetaSystems Probes GmbH, Altussheim, Germany), which are CE-labeled and classified as IVD products in the EU according to the In Vitro Diagnostic Medical Device Directive 98/79/EC. Samples had been first tested with the probes 1p32.3/1q21-22 (CDKN2C/CKS1B), 8q24 (MYC; break apart), 13q14.2/17p13 (DLEU/TP53), and 14q32.3 (IGH; break apart). If a split signal had been detected with the IGH break apart probe, additional testing with the Translocation/Dual Fusion probes 4p16.3/14q32.3 (FGFR3/IGH), 11q13.3/14q32.3 (MYEOV/IGH), 14q32.3/16q23 (IGH/MAF), 6p21.1/14q32.3 (CCND3/IGH), and 14q32.3/20q12 (IGH/MAFB) had been performed according to the international recommendations [8].

### Statistical analysis

Absolute numbers (percentages) are given to describe categorical variables. Continuous variables are depicted by the median (interquartile range, IQR). Percent BMPC values below the detection limits were replaced by half of the smallest value actually measured (i.e., the value 1 in case of hBMPC (13 times); the value 0.5 in case of sBMPC (9 times); the value 0.05 in case of fBMPC (3 times)). Differences between the percent BMPC measurements of the three methods are illustrated using Bland-Altman plots [9], and the nonparametric Wilcoxon signed rank test was calculated for statistical comparisons. To evaluate the effect of preparative and cell-specific features on the magnitude of the percent BMPC differences, the nonparametric Wilcoxon rank sum test (2-group comparisons) and the nonparametric Kruskal-Wallis test (> 2 groups) were used. Since 2*21 statistical tests were performed to explore this issue, the Bonferroni correction was applied to adjust for the number of multiple comparisons, and therefore, only two-sided *P* values < 0.0012 (= 0.05/42) were considered as indicating statistical significance. Cohen’s kappa coefficient (*κ*) is given to describe the degree of agreement between the categorized percent BMPC values. The nonparametric Wilcoxon signed rank test was applied to compare measurements using the Ficoll method with the measurements with the RBC lysis method.

## Results

### Differences between methods

**Fig. 1:**
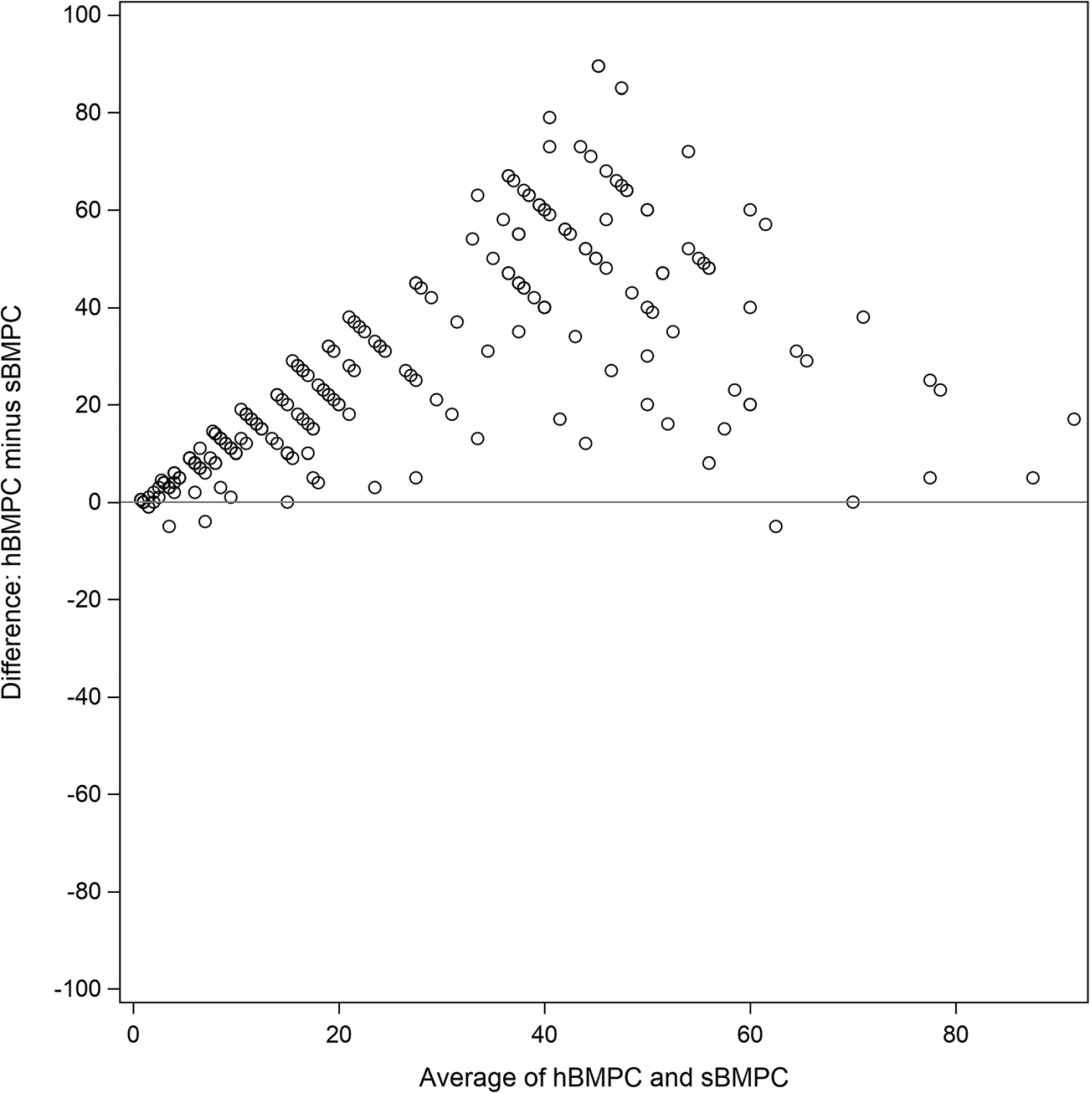
Bland-Altman plot. Differences of bone marrow plasma cell (BMPC) numbers between histomorphologic (h) count and smear (s) count

**Fig. 2:**
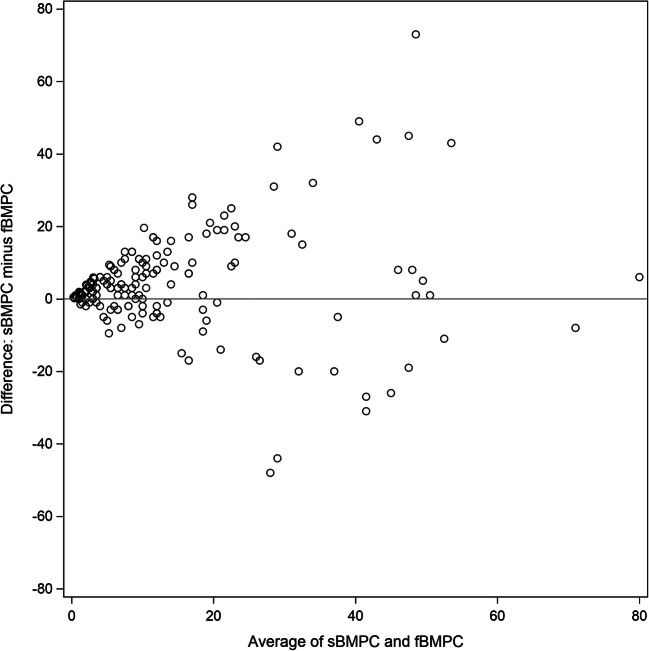
Bland-Altman plot. Differences of bone marrow plasma cell (BMPC) numbers between smear (s) count and flow cytometry (f) count

The median (IQR) proportion of estimated BMPC was 30.0% (15.0–70.0%) by histological review (hBMPC), 7.0% (2.0–16.0%) by smear review (sBMPC), and 3.0% (0.8–10.0%) by flow cytometry (fBMPC). Numbers of hBMPC were nearly always higher than the respective values counted on aspirate smears (median difference 19.5 percentage points (pp) (IQR 8.0–43.0 pp); *P* < 0.0001; Fig. 1). The magnitude of the difference between hBMPC and sBMPC was independent of the quality score of the core biopsy specimen (Table 1). The difference between sBMPC and fBMPC was also significant (median difference 1.9 pp (0.2–6.0 pp); *P* < 0.0001). In 75% (179/238) of cases, the recovery of sBMPC was better than that of fBMPC; 25% of cases exhibited equal (*n* = 13) or even superior (*n* = 46) recovery of fBMPC (Fig. 2). The latter group included 8 cases in which the aspirate smear was not adequate.

**Table 1:** Effects of preparative and cell-specific features on differences in plasma cell numbers

Variables		hBMPC versus sBMPC, difference, %		sBMPC versus fBMPC, difference, %	
	No.	Median (IQR)	*P* value	Median (IQR)	*P* value
Referring institution					
MUV 1	76	24 (9.5–46)	0.312	2 (0.4–8)	0.526
MUV 2	61	18 (9–40)		1.6 (0.4–4)	
MUV 3	34	14.3 (6–22)		1.3 (0.5–3.7)	
Extern	67	20 (8–42)		3 (− 1–10)	
Quality of smear					
Adequate	226	66.5 (50–76)	< 0.0001*	2 (0.5–7)	0.0007*
Not adequate	12	18 (8–40)		− 2 (− 12.8–0.4)	
Quality of biopsy					
Very good/good	206	18.0 (8–40)	0.242		
Moderate/poor	32	28.0 (10–48)			
Plasma cell atypia					
Absent/low	140	15 (8–30)	< 0.0001*	1.8 (0.8–4)	0.263
Moderate/high	91	33 (15–52)		4 (− 4–11)	
Bone marrow fibers					
Not increased	188	17.5 (7.5–36)	< 0.0001*	2 (0.5–7)	0.002
Increased	40	51 (14.5–64)		0.5 (− 4.5–3)	
Amyloid					
Absent	200	20 (8–45)	0.880	1.8 (0–7)	0.650
Present	29	17 (11–29)		2 (1–4.7)	
Clonality all					
Kappa	124	26 (13–47)	< 0.0001*	3 (0–9.7)	0.027
Lambda	91	18 (9–44)		1.8 (0–4)	
Polyclonal	23	3 (0–6)		0.9 (0.8–1.6)	
Clonality < 10% sBMPC					
Kappa	73	19 (10–33)	< 0.0001*	1 (0–3)	0.656
Lambda	62	13 (9–28)		1.7 (0–3)	
Polyclonal	22	3 (0–6)		0.9 (0.8–1.6)	
Surface immunophenotype					
CD19+	25	4 (0.5–8)	< 0.0001*	1.0 (0.8–3)	0.270
CD19−	212	22 (10–45)		2 (0–7.5)	
CD56+	134	26.5 (13–48)	< 0.0001*	3 (0.2–10)	< 0.0001*
CD56−	104	13 (5–28.5)		1 (0.1–3)	
CD117+	69	28 (13–47)	0.010	3 (1–10)	0.003
CD117−	168	17 (6.5–37.5)		1.6 (0–4.9)	
CD20+	21	18 (13–35)	0.800	2 (0.2–6)	0.654
CD20−	212	20 (8–42.5)		1.8 (0.3–7)	
Cytogenetic aberrations					
*TP53* del./yes	22	41 (17–52)	0.036	1.5 (− 8–7)	0.159
*TP53* del./no	163	21 (10–40)		2 (0.9–8)	
1p del./yes	15	38 (18–43)	0.156	6 (− 2–17)	0.550
1p del./no	168	21.5 (10–45)		2.1 (0.7–7)	
1q gain/yes	60	31.5 (15–56)	0.005	2 (− 1.5–8.5)	0.788
1q gain/no	123	20 (10–36)		3 (0.8–7)	
*IgH* abnorm/yes	109	25 (13–45)	0.099	2.2 (0–6)	0.704
*IgH* abnorm/no	76	18.5 (9–41)		2 (0.3–8)	
t(4;14)/yes	16	27.5 (6.5–49)	0.777	1.6 (− 4–6.5)	0.534
t(4;14)/no	79	23 (15–47)		2 (0–7)	
t(11;14)/yes	48	20.5 (14–37)	0.101	2 (0–6)	0.444
t(11;14)/no	46	31.5 (16–56)		3 (0–11)	
*MYC* abnorm/yes	39	40 (20–60)	0.0002*	5 (− 3–10)	0.337
*MYC* abnorm/no	144	19.5 (10–38)		2 (0.5–6)	
13q del./yes	82	29.5 (15–50)	0.027	3 (− 2–8)	0.835
13q del./no	101	20 (9–38)		2 (0.8–7)	
Sum of cytogenetic aberrations					
0	29	13 (8–23)	0.0002*	1.8 (0–3)	0.229
1–3	114	20 (10–45)		3 (1–8)	
> 3	40	38.5 (23–56)		3 (− 4–9.5)	

*BMPC*, bone marrow plasma cells; *hBMPC*, enumerated by histology review; *sBMPC*, enumerated by smear review; *fBMPC*, enumerated by flow cytometry; *MUV*, Medical University of Vienna (1, Division of Hematology and Hemostaseology; 2, Division of Oncology; 3, Division of Nephrology and Dialysis); *del*, deletion; *abnorm,* abnormality

^*^Statistically significant after Bonferroni correction for multiple comparisons

### Impact of cell-specific characteristics

Details on preparative and BMPC-specific characteristics and their effects on the differences between hBMPC and sBMPC as well as between sBMPC and fBMPC are listed in Table 1.

#### Core biopsy versus aspirate smear

The disparity of results between core biopsy and aspirate smear count was significantly enhanced in case of inadequate quality of the smear (*P* < 0.0001), higher-grade cell atypia (*P* < 0.0001), increased BM fiber content (*P* < 0.0001), and expression of CD56 (*P* < 0.0001). Furthermore, the difference was associated with abnormal FISH signal patterns for the MYC probe indicating rearrangement or gain of *MYC* (*P* = 0.0002), as well as with the number of cytogenetic aberrations (*P* = 0.0002) (Table 1). Conversely, expression of CD19 and a non-clonal plasma cell phenotype were significantly associated with a lower difference between numbers of hBMPC and sBMPC (both *P* < 0.0001). This association remained statistically significant in a subgroup analysis including only samples with ≤ 10% sBMPC (*n* = 157; *P* < 0.0001) (Table 1).

#### Aspirate smear versus flow cytometry

The difference between the percentages of sBMPC and fBMPC was significantly associated with the quality of the smear (*P* = 0.0007) and expression of CD56 (*P* < 0.0001) (Table 1).

### Impact on disease classification and assessment of treatment response

Results of morphological methods were applied to the threshold levels designating plasma cell myeloma (10% and 60% BMPC; Table 2) and treatment response (5% BMPC; Table 3). In 108/145 (74%) cases that exhibited < 10% BMPC on the aspirate smear, the percentage of BMPC was ≥ 10% on histomorphologic examination. In addition, more than half of cases (44/86) with aspirate smear levels between 10 and 60% already exhibited > 60% BPMC in the core biopsy specimen. Overall agreement between hBMPC and sBMPC was very low (weighted kappa, *κ* = 0.18; 95% confidence interval (CI) [0.12–0.23]). Eighty-four of 101 (83%) cases with < 5% BMPC on the aspirate smear exhibited ≥ 5% BMPC on the core biopsy count. Again, agreement was very low (*κ* = 0.18; 95% CI [0.10–0.26]).

**Table 2:** Comparison of hBMPC and sBMPC counts with regard to disease classification

% sBMPC	% hBMPC			
	< 10	10–60	> 60	Total
< 10	37	91	17	145
10–60	0	42	44	86
> 60	0	1	6	7
Total	37	134	67	238

*BMPC*, bone marrow plasma cells; *hBMPC*, enumerated by histology review; *sBMPC*, enumerated by smear review

**Table 3:** Comparison of hBMPC and sBMPC counts with regard to assessment of complete response

% sBMPC	% hBMPC		
	< 5	≥ 5	Total
< 5	17	84	101
≥ 5	1	136	137
Total	18	220	238

*BMPC*, bone marrow plasma cells; *hBMPC*, enumerated by histology review; *sBMPC*, enumerated by smear review

### Density gradient centrifugation versus red cell lysis for flow cytometry

To evaluate the influence of sample preparation on the recovery of fBMPC, we carried out a separate prospective analysis comparing our routinely used Ficoll method with the RBC lysis method. The Ficoll method yielded a median of 1.3% (0.2–14.0%) of BMPC, whereas the lysis method recovered 0.5% (0.1–4.0%) of BMPC (median difference 0.4 pp (0.05–1.5 pp) *P* = 0.0001). Detailed results are shown in Table 4. The Ficoll method was superior in 32/40 cases, equal results were obtained in 2 cases, and in 6 cases, the lysis method recovered more plasma cell events. Among the 24 cases with smear counts of ≤ 5% BMPC, the Ficoll method yielded superior counts in 20 (83%) cases.

**Table 4:** BMPC numbers recovered after Ficoll and RBC lysis preparation of flow cytometry samples*

Case no.	sBMPC (%)	fBMPC (%)	
		Ficoll	RBC lysis
1	37	16	*23*
2	1	0.1	0.1
3	6	*12*	3
4	1	*0.1*	< 0.1
5	2	*0.6*	0.1
6	1	*0.1*	< 0.1
7	< 1	*0.2*	0.1
8	1	*0.2*	0.1
9	< 1	*0.1*	< 0.1
10	2.5	0.2	*0.5*
11	4	*1.4*	0.6
12	1	*1.1*	0.2
13	6	2.1	*2.6*
14	11	*1.5*	1.3
15	22	*26*	13.5
16	1.5	2.7	*2.8*
17	3	*0.7*	0.1
18	1	*0.4*	< 0.1
19	1.5	*1*	0.2
20	20	*2.7*	0.5
21	2	*0.3*	0.1
22	57	*32*	13
23	57	*36*	23
24	27	*16*	5
25	1	*0.2*	< 0.1
26	35	29	*36*
27	8	*0.8*	0.2
28	5	*5.2*	2.6
29	55	25	*28*
30	4	*2.2*	1.6
31	< 1	*0.3*	0.2
32	40	*60*	23
33	2	*2.5*	1.2
34	1	0.2	0.2
35	4.5	*0.2*	< 0.1
36	65	*37*	36
37	1.5	*0.2*	0.1
38	40	*32*	18
39	5	*1*	0.4
40	9	*2.5*	0.8

Numbers in italics script indicate the higher value obtained by the respective comparison

*BMPC*, bone marrow plasma cells; *sBMPC*, enumerated by smear review; *fBMPC*, enumerated by flow cytometry; *RBC*, red blood cell

^*^The difference was statistically significant *P* < 0.0001

## Discussion

The classification of plasma cell disorders requires precise estimation of the percentage of BMPC [1]. In the present study, we show that the two morphological methods that are commonly used to determine the content of BMPC at diagnosis yield divergent results. Plasma cell percentages obtained by estimates from core biopsies were substantially higher than plasma cell percentages obtained by counts from aspirate smears, irrespective of the quality of the biopsy specimen. This is a well-known phenomenon, and it is therefore recommended to use the higher plasma cell value for diagnosis in cases where both methods have been applied [2, 3, 5, 6, 10–15]. In the present study, observed differences between numbers of hBMPC and sBMPC are of major clinical relevance. In fact, 74% of cases that would have been classified as MGUS by smear morphology already fulfilled the criteria for plasma cell myeloma on histomorphologic examination. Similar rates of misclassification of MGUS by aspirate smear counts have also been observed by others [12, 13]. At the threshold level of ≥ 60% plasma cells on the core biopsy, more than half of our cases would have been missed by the smear count. Criteria to define complete response after treatment of myeloma include reduction of BMPC content to < 5% [16, 17]. In the present study, 83% of cases with < 5% of BMPC on the aspirate smear would not fulfill this criterion by histologic examination. These data strongly suggest that the percentage of BMPC on the aspirate smear is not a safe diagnostic indicator of plasma cell myeloma. The diagnostic impact of cytomorphology rather focusses on the identification of dysplastic and/or immature plasma cells as indicators of malignant disease. Accordingly, in the present study, higher-grade cell atypia on cytomorphologic examination was associated with a significantly higher difference between percentages of sBMPC and hBMPC.

Possible explanations for the observed differences between BMPC recovery in biopsy specimens and aspirate smears include dilution of aspirates with peripheral blood as well as aspiration failure due to fibrotic bone marrow environment or increased niche adherence of malignant plasma cells [10]. In the present study, inadequate cellularity of the aspirate specimen and increased BM fiber content were significantly correlated with a lower yield of sBMPC. Moreover, aberrant expression of CD56 on plasma cells was associated with a significantly higher difference between numbers of hBMPC and sBMPC, and further, between numbers of sBMPC and fBMPC. The CD56 molecule is a cell adhesion molecule, which is aberrantly expressed in the majority of plasma cell myelomas [18, 19]. It has been postulated that overexpression of CD56 on myeloma cells forces their adherence to stroma cells [20]. This hypothesis is in line with the clinical observation that the downregulation of CD56 on myeloma cells is associated with extramedullary spreading of the disease [20–22]. The present study adds another clinically relevant aspect to the adhesive role of CD56. We demonstrate that CD56 expressing BMPC are more difficult to aspirate than BMPC that lack CD56 and, thus, disclose a potential bias for the assessment of MRD after therapy. As methods to detect and quantify MRD rely on bone marrow aspirate specimens, aspiration failure of CD56 expressing BMPC may lead to underestimation of the actual amount of residual myeloma cells. Our data strongly support the recommendation to use first pull aspirates for the assessment of MRD [7].

We also found that features of a non-malignant plasma cell phenotype were associated with a significantly lower difference between numbers of hBMPC and sBMPC, indicating that normal, non-clonal BMPC might more easily be aspirated than clonal plasma cells. This hypothesis is supported by the clinical observation that in non-malignant reactive conditions, substantial amounts of plasma cells can be seen on blood smears, whereas in multiple myeloma irrespective of the degree of bone marrow infiltration, usually no or only low amounts of plasma cells are found in the peripheral blood [23, 24]. It has been shown that the proportion of clonal plasma cells within the total BMPC compartment has prognostic significance in terms of risk of progression of MGUS and smoldering myeloma as well as in terms of predicting risk of relapse after stem cell transplantation [25, 26]. We therefore performed a subanalysis including only cases with ≤ 10% BMPC on the aspirate smear, which confirmed the better recovery of non-clonal plasma cells on smear preparations. Accurate estimation of the ratio between non-clonal and clonal BMPC is only possible from bone marrow aspirates. If, however, clonal plasma cells can less easily be pulled out than their non-clonal counterparts, the measured ratio within an aspirate sample might underestimate the actual proportion of clonal cells.

It has been shown that circulating myeloma cells may have a different genetic profile than their BM counterparts [22]. In the present study, multiple genetic aberrations as well as abnormalities of the *MYC*-gene were associated with a lower BMPC recovery on aspirate smears, supporting the idea that adherence of plasma cells to BM may also be determined by their genetic background. Aspiration failure of certain myeloma (sub) clones might, as well, represent a possible bias to the detection of residual abnormal cells.

It is commonly recognized that BMPC are vastly under-represented by flow cytometry [4, 27–31]. We also found that flow cytometry yielded significantly lower amounts of BMPC compared with smear morphology, which further confirms that it is the least reliable method to discriminate between plasma cell myeloma and MGUS. However, in the present study, the difference between sBMPC and fBMPC was not as pronounced as reported by others, and in about one-fifth of cases, it was even reverse. In part, this applied to cases with inadequate cellularity of the bone marrow smear. We further questioned whether the type of sample preparation might influence the flow cytometric recovery of plasma cells. Basically, there are two different ways to prepare BMPC for flow analysis. With the RBC lysis method, whole bone marrow is processed after adding a red cell lysing agent. With the Ficoll method, the mononuclear fraction of bone marrow cells is processed after density gradient centrifugation. Our results were obtained with the Ficoll method, whereas most of the formerly reported studies had used the RBC lysis method. Concern has been raised that density gradient centrifugation may unpredictably result in enrichment or loss of BMPC [4]*.* However, definitive data to confirm this assumption are lacking. We therefore conducted a prospective follow-up investigation to compare the two methods and found that the Ficoll method yielded more BMPC events in 80% of investigated cases. The superiority of the Ficoll method was even more pronounced in cases with low level BMPC. These results do not argue in favor of a role of the Ficoll method as a disease classification tool, as cytomorphology still recovered more BMPC in the majority of cases. However, our findings may encourage further investigations to find out whether the Ficoll method is in fact more effective in recovering minimal amounts of BMPC after therapy, which is the core competence of flow cytometry in the management of plasma cell myeloma.

In conclusion, our study sheds new light on a known phenomenon. It is commonly recognized that the core biopsy count is the most reliable method to estimate the percentage of BMPC. Nevertheless, the current criteria for classification of plasma cell disorders do not definitely claim a core biopsy count for BMPC enumeration [1]. We show that the aspiration failure of BMPC is not only a matter of sampling quality, but also dependent on cell-specific features. Thus, even in good quality aspirate smears, the plasma cell content may significantly differ from the actual proportion in situ. Consequently, the core biopsy must be regarded indispensable for correct disease classification and evaluation of complete response. Furthermore, reports of aspirate smear counts should include a note that indicates the need for a core biopsy count to definitely quantify the plasma cell infiltration. Aspiration failure due to malignant type features of BMPC may also bias the flow cytometric detection of minimal amounts of residual myeloma cells. Efforts to enrich BMPC in flow cytometry samples may at least in part compensate for this deficit.
